# 714 Actinomyces Necrotizing Soft Tissue Infections: A Case Series from a Single Institution

**DOI:** 10.1093/jbcr/irad045.190

**Published:** 2023-05-15

**Authors:** Aislinn Lewis, Ann Marie Prazak, Callie Thompson

**Affiliations:** University of Utah, Salt Lake City, Utah; University of Utah, Salt Lake City, Utah; University of Utah, Salt Lake City, Utah; University of Utah, Salt Lake City, Utah; University of Utah, Salt Lake City, Utah; University of Utah, Salt Lake City, Utah; University of Utah, Salt Lake City, Utah; University of Utah, Salt Lake City, Utah; University of Utah, Salt Lake City, Utah

## Abstract

**Introduction:**

Necrotizing soft tissue infections (NSTI) are rare, severe infections that can lead to septic shock. NSTI have a mortality rate of 20-50% in patients who require intensive care. Management includes surgical debridement and broad-spectrum antibiotics until definitive culture results. Actinomyces is a slow-growing bacterium found in the mouth, skin, genitourinary, and gastrointestinal tracts, with several case reports of actinomyces related NSTI. At this single institution, NSTI are managed by burn surgeons. The goal of this study is to quantify patients with actinomyces-related infections, identify modifiable risk factors, and demonstrate the pharmacology of effective antibiotic treatment.

**Methods:**

All patients with actinomyces-related infections admitted to a single institution’s burn center from January 2020 to September 2022 were included. Patients were identified via the institution’s data warehouse. Patient demographics, infection location, associated injuries or illnesses, antibiotic exposure, and treatments required were obtained. Results were recorded and compared using descriptive statistical analysis.

**Results:**

A total of 22 patients with actinomyces infections were identified, 18 with NSTI and 4 with non-NSTI infections (1 hidradenitis suppurativa, 1 Job’s syndrome, and 2 burn-related). Average age was 50.3 years old, and 68% of patients were male. Seventy-four percent of patients were obese, and average BMI for obese patients was 40.3 kg/m^2^. Fourteen NSTI patients had diabetes or pre-diabetes (mean A1c 9.4%), and two were taking a sodium-glucose co-transporter 2 inhibitor. All NSTI patients underwent surgical debridement and broad-spectrum antibiotic treatment. The groin was the most common site of infection. Time from collection of sample to culture resulting with Actinomyces was 44 to 185 hours (mean 111 hours). Species isolated were A. europaeus, A. radingae, A. turicensis, A. odontolyticus, and A. funkei. Three of the NSTI patients died. Among NSTI survivors, average length of stay was 34 days, duration of antibiotics was 10.7 days (6 to 16 days), and 50% of patients were de-escalated to either ampicillin/sulbactam, or ertapenem.

**Conclusions:**

To our knowledge this is the largest single institutional series to date for patients affected by Actinomyces related NSTI. The high percentage of patients with diabetes, 73% of NSTI patients, suggests an increased susceptibility to infection for these patients. A variety of Actinomyces isolates are present in NSTI. The mortality rate (17%) of these ICU admitted NSTI patients agrees with prior studies.

**Applicability of Research to Practice:**

Novel findings of actinomyces infection in NSTI patients

